# Host Poly(A) Polymerases PAPD5 and PAPD7 Provide Two Layers of Protection That Ensure the Integrity and Stability of Hepatitis B Virus RNA

**DOI:** 10.1128/JVI.00574-21

**Published:** 2021-08-25

**Authors:** Fei Liu, Amy C. H. Lee, Fang Guo, Andrew S. Kondratowicz, Holly M. Micolochick Steuer, Angela Miller, Lauren D. Bailey, Xiaohe Wang, Shuai Chen, Steven G. Kultgen, Andrea Cuconati, Andrew G. Cole, Dimitar Gotchev, Bruce D. Dorsey, Rene Rijnbrand, Angela M. Lam, Michael J. Sofia, Min Gao

**Affiliations:** a Arbutus Biopharma, Warminster, Pennsylvania, USA; University of Southern California

**Keywords:** RNA integrity, RNA stability, PAPD5, PAPD7, AB-452, HBV PRE, HBV RNA integrity

## Abstract

Noncanonical poly(A) polymerases PAPD5 and PAPD7 (PAPD5/7) stabilize hepatitis B virus (HBV) RNA via the interaction with the viral posttranscriptional regulatory element (PRE), representing new antiviral targets to control HBV RNA metabolism, hepatitis B surface antigen (HBsAg) production, and viral replication. Inhibitors targeting these proteins are being developed as antiviral therapies; therefore, it is important to understand how PAPD5/7 coordinate to stabilize HBV RNA. Here, we utilized a potent small-molecule AB-452 as a chemical probe, along with genetic analyses to dissect the individual roles of PAPD5/7 in HBV RNA stability. AB-452 inhibits PAPD5/7 enzymatic activities and reduces HBsAg both *in vitro* (50% effective concentration [EC_50_] ranged from 1.4 to 6.8 nM) and *in vivo* by 0.94 log_10_. Our genetic studies demonstrate that the stem-loop alpha sequence within PRE is essential for both maintaining HBV poly(A) tail integrity and determining sensitivity toward the inhibitory effect of AB-452. Although neither single knockout (KO) of *PAPD5* nor *PAPD7* reduces HBsAg RNA and protein production, *PAPD5* KO does impair poly(A) tail integrity and confers partial resistance to AB-452. In contrast, *PAPD7* KO did not result in any measurable changes within the HBV poly(A) tails, but cells with both *PAPD5* and *PAPD7* KO show reduced HBsAg production and conferred complete resistance to AB-452 treatment. Our results indicate that PAPD5 plays a dominant role in stabilizing viral RNA by protecting the integrity of its poly(A) tail, while PAPD7 serves as a second line of protection. These findings inform PAPD5-targeted therapeutic strategies and open avenues for further investigating PAPD5/7 in HBV replication.

**IMPORTANCE** Chronic hepatitis B affects more than 250 million patients and is a major public health concern worldwide. HBsAg plays a central role in maintaining HBV persistence, and as such, therapies that aim at reducing HBsAg through destabilizing or degrading HBV RNA have been extensively investigated. Besides directly degrading HBV transcripts through antisense oligonucleotides or RNA silencing technologies, small-molecule compounds targeting host factors such as the noncanonical poly(A) polymerase PAPD5 and PAPD7 have been reported to interfere with HBV RNA metabolism. Herein, our antiviral and genetic studies using relevant HBV infection and replication models further characterize the interplays between the *cis* element within the viral sequence and the *trans* elements from the host factors. PAPD5/7-targeting inhibitors, with oral bioavailability, thus represent an opportunity to reduce HBsAg through destabilizing HBV RNA.

## INTRODUCTION

Globally, more than 250 million patients are chronically infected with hepatitis B virus (HBV) (44), but a functional cure of chronic hepatitis B (CHB) is rarely achieved even after years of treatment with nucleos(t)ide analogues (NAs) such as entecavir (ETV) and tenofovir disoproxil fumarate (TDF) (1). Pegylated interferon alpha (IFN-α) enhances antiviral immune response, but the cure rate remains low, and side effects are often difficult to tolerate (2, 3). The major obstacles to curing CHB include the persistence of the episomal covalently closed circular DNA (cccDNA) and an immune system that is tolerized to HBV, likely due to the excess amount of circulating hepatitis B surface antigen (HBsAg) levels (4–6).

The HBV envelope proteins preS1, preS2, and HBsAg are synthesized in the endoplasmic reticulum and are secreted as both viral and subviral particles (7, 8). HBV virions are double-shelled particles with an outer lipoprotein bilayer containing the envelope proteins and an inner nucleocapsid that encloses the HBV DNA and viral polymerase. The subviral particles devoid of nucleocapsids and HBV DNA (9, 10) are up to 100,000-fold in excess relative to the virions in the blood of infected patients (11). Such high levels of subviral particles are believed to play a key role in immune tolerance and maintenance of persistent HBV infection (5, 6). In patients with chronic hepatitis B, HBV-specific T cells are depleted or functionally impaired (12–15), and circulating and intrahepatic antiviral B cells are defective in the production of antibodies against HBsAg, with an expansion of atypical memory B cells (16, 17). HBsAg has also been linked to the inhibition of innate immunity and functionality of other immune cell types (18). Therefore, antiviral strategies that aim to target the HBV RNA transcripts could suppress HBsAg production and may break the immune tolerance state to potentially increase the functional cure rate.

Regulation of HBV RNA metabolism involves the posttranscriptional regulatory element (PRE), which is a stretch of ribonucleotides spanning positions 1151 to 1582 on the viral transcripts that is essential to HBV subgenomic RNA (sRNA) nuclear export and regulation of pregenomic RNA (pgRNA) splicing (19–22). The PRE contains three subelements, PREα, PREβ1, and PREβ2. Each subelement is sufficient to support sRNA nuclear export and HBsAg production, but all three together exhibit much greater activity (23, 24). RNA secondary structure prediction and phylogenetic covariations analysis suggest that two stem-loop structures (stem-loop alpha [SLα] and SLβ1) localized either in PREα or PREβ1 subelements may exist *in vivo* and serve as protein binding sites (23, 25). These two stem-loop structures are highly conserved not only in HBV variants but also throughout the different mammalian hepadnaviruses, and mutations in the stem regions reduced HBsAg production (23). The PRE is complexed with several RNA binding proteins, including T-cell intracellular antigen 1, La protein, polypyrimidine tract binding protein, ZC3H18, and ZCCHC14 (26–32). These PRE binding proteins may serve to regulate the export and stability of HBV RNAs. In particular, the CAGGC pentaloop sequence/structure of SLα within the PREα subelement has been predicted to bind sterile alpha motif domain-containing proteins (24). Recently, ZCCHC14 (a sterile alpha motif-containing protein), together with PAPD5 and PAPD7 [the noncanonical poly(A) RNA polymerase-associated domain-containing proteins 5 and 7], were identified as the cellular binding proteins that interacted with the HBV SLα sequence (33).

The small-molecule compound, RG7834, targets PAPD5/7 and destabilizes HBV RNAs (34–37). Using a genome-wide CRISPR screen, it was subsequently observed that *ZCCHC14* and *PAPD5* were associated with the antiviral activity of RG7834 (32). Interestingly, individual knockdown of *PAPD5* or *PAPD7* had minimal effect against HBsAg production, while knockdown of *ZCCHC14* or double knockdown of *PAPD5*/*7* had a profound anti-HBsAg activity similar to that observed when cells were treated with RG7843 (32, 37). It was further demonstrated that double knockout of *PAPD5/7* reduced guanosine incorporation frequency within HBV RNA poly(A) tails, leading to a proposed model in which HBV RNA recruits the PAPD5/7-ZCCHC14 complex via the CNGGN pentaloop of PRE SLα to enable the extension of mixed tailing on HBV poly(A) tails, which subsequently protects the viral RNAs from cellular poly(A) ribonucleases (33).

To gain further insights into how small-molecule inhibitors destabilize HBV RNAs, mechanistic studies were performed using AB-452, an analogue of RG7834, to evaluate its effect in HBV-replicating cells and in cells transfected with constructs containing mutations within the PRE sequence. To better understand how PAPD5 and PAPD7 coordinate in the protection of HBV RNAs, both HBV RNA transcripts and their poly(A) tails were analyzed in cells with *PAPD5* and/or *PAPD7* knockout. Our results reveal that HBV utilizes two layers of protection mechanism provided by PAPD5 and PAPD7 to protect their poly(A) tail integrity and RNA stability.

## RESULTS

### AB-452 inhibits HBV *in vitro* and *in vivo*.

AB-452 and RG7834 both belong to the dihydroquinolizinones chemical class. The antiviral activities of AB-452 and its diastereomer ARB-169451 were evaluated using multiple *in vitro* HBV replication models, including HepG2.2.15 cells (which constitutively express HBV through the integrated viral genome), PLC/PRF/5 cells (a patient-derived hepatocellular carcinoma cell line only expressing HBsAg), and HBV-infected HepG2-NTCP cells or primary human hepatocytes (PHHs) in which viral replication was dependent on cccDNA transcription (Table 1). AB-452 reduced HBsAg, HBeAg, and HBV DNA production with 50% effective concentration (EC_50_) values ranging from 0.28 to 6.8 nM, while its diastereomer ARB-169451 was more than 1,000-fold weaker toward HBsAg inhibition than AB-452 (Table 1). AB-452 antiviral activity was specific for HBV, as the compound was inactive against a panel of 10 different RNA and DNA viruses with EC_50_ values of >30 μM (Table 2). In addition, the cytotoxicity of AB-452 was evaluated in several cell lines from different tissue origins showing 50% cytotoxic concentration (CC_50_) values of >30 μM (the highest concentration tested) (Table 3), demonstrating the selectivity of AB-452.

**TABLE 1:**
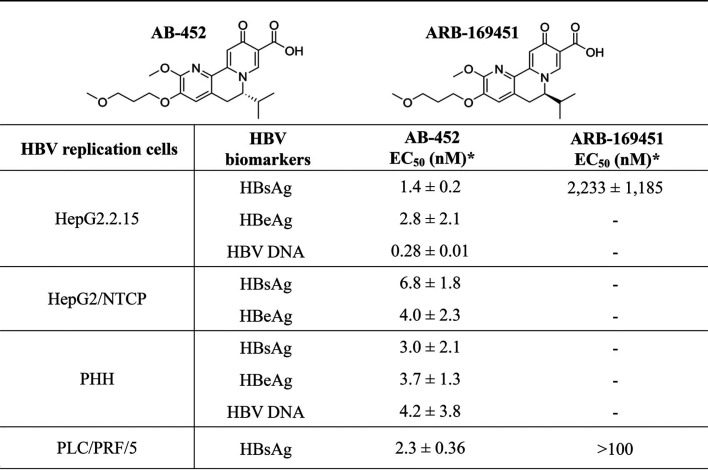
*In vitro* anti-HBV effect of AB-452 and ARB-169451^*a*^

a Asterisks represent mean EC_50_s ± standard deviations that were determined from at least three independent experiments.

**TABLE 2:** Antiviral selectivity of AB-452 against a panel of DNA and RNA viruses^*a*^

Virus	Cell line	Measurement type	AB-452 EC_50_ (μM)
HBV	HepG2-2.2.15	HBsAg	0.0012
HSV-1/HSV-2	Vero	CPE	>30
HCMV	MRC-5	CPE	>30
HIV	CEM-SS	CPE	>30
HCV	Huh-7	Luciferase	>30
DENV2	BHK21	CPE	>30
WNV	Vero76	CPE	>30
RSV	HEp2	CPE	>30
Human rhinovirus	H1-HeLa	CPE	>30
Influenza	MDBK	CPE	>30

a CPE, cytopathic effect; HSV-1, herpes simplex virus 1; HCMV, human cytomegalovirus; HCV, hepatitis C virus; DENV2, Dengue virus 2; WNV, West Nile virus; RSV, respiratory syncytial virus.

**TABLE 3:** Cytotoxicity of AB-452 against cells derived from various tissues

Cell line	Tissue origin	AB-452 CC_50_ (μM)
HepG2-2.2.15	Liver	>30
Huh-luc/neo-ET	Liver	>30
CEM-SS	T lymphoblast	>30
Vero	Kidney	>30
MDBK	Kidney	>30
MRC5	Lung fibroblasts	>30
HEp2	Human epithelial cells	>30
HeLa	Human cervical cancer cells	>30
BHK21	Kidney	>30

**FIG 1:**
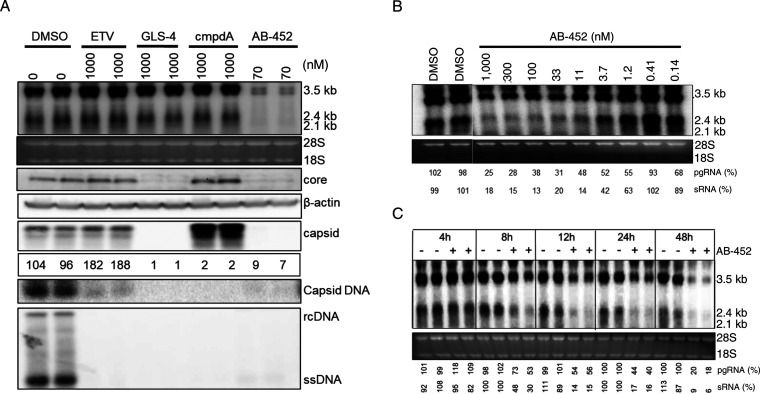
AB-452 interferes with multiple steps of HBV life cycle by reducing HBV RNA. (A) HepG2.2.15 cells were treated with DMSO, ETV (1 μM), GLS-4 (1 μM), cmpdA (1 μM), or AB-452 (70 nM) for 6 days. Intracellular HBV pgRNA (3.5 kb) and sRNA (2.4 kb and 2.1 kb) were analyzed by Northern blotting. Intracellular core protein was detected by Western blotting. Intracellular HBV capsids were detected by the capsid particle gel assay based on agarose gel electrophoresis. Intracellular HBV DNA was detected by Southern blotting. Encapsidated pgRNA (capsid pgRNA) was quantitated by reverse transcription-quantitative PCR (qRT-PCR) and expressed as percentage of untreated controls (DMSO). rRNA (18S and 28S) and β-actin serve as loading controls for analysis of HBV RNA and core protein, respectively. (B) Levels of intracellular pgRNA and sRNA in HepG2.2.15 cells treated with increasing concentrations of AB-452 (0.14 to 1,000 nM) for 48 h. Percentages of pgRNA and sRNA were determined by normalizing to untreated controls. (C) Time course analysis of HBV RNAs from cells treated with and without 70 nM AB-452. Total intracellular RNA was extracted from cells harvested at 4-, 8-, 12-, 24-, and 48-h time points posttreatment. HBV pgRNA and sRNA were analyzed by Northern blotting. Percentages of pgRNA and sRNA were determined by normalizing to untreated controls at each time point.

To evaluate the effects of AB-452 against the different stages of the viral life cycle, HBV replication intermediates and viral proteins were analyzed from HepG2.2.15 cells treated with AB-452 at a concentration of 50-fold above its EC_50_ value (Fig. 1A). The nucleoside analog ETV and two classes of HBV capsid inhibitors, GLS-4 (class I) and compound A (cmpdA, class II), were included as controls (structures not shown) targeting the polymerase and core/capsid proteins, respectively. ETV strongly inhibited HBV DNA replication but did not reduce the production of intracellular HBV RNA, core protein levels, or capsid assembly. Consistent with their mechanism of action, capsid inhibitors inhibited pgRNA encapsidation and HBV DNA replication but had no effect against total pgRNA and sRNA transcripts. On the contrary, AB-452 displayed a unique antiviral phenotype reducing intracellular pgRNA, sRNA, core protein, native capsids, encapsidated pgRNA, and replicating HBV DNA (Fig. 1A). Furthermore, the effect of AB-452 against intracellular pgRNA and sRNA was dose dependent and appeared to reach a plateau at >100 nM, with approximately 25% pgRNA and 18% sRNA remaining detectable at the highest concentration tested (1 μM) (Fig. 1B). Results from the time course studies showed that AB-452 induced reduction of pgRNA and sRNA starting at 8 h posttreatment, and the levels continued to decline through the 48-h treatment period (Fig. 1C).

**FIG 2:**
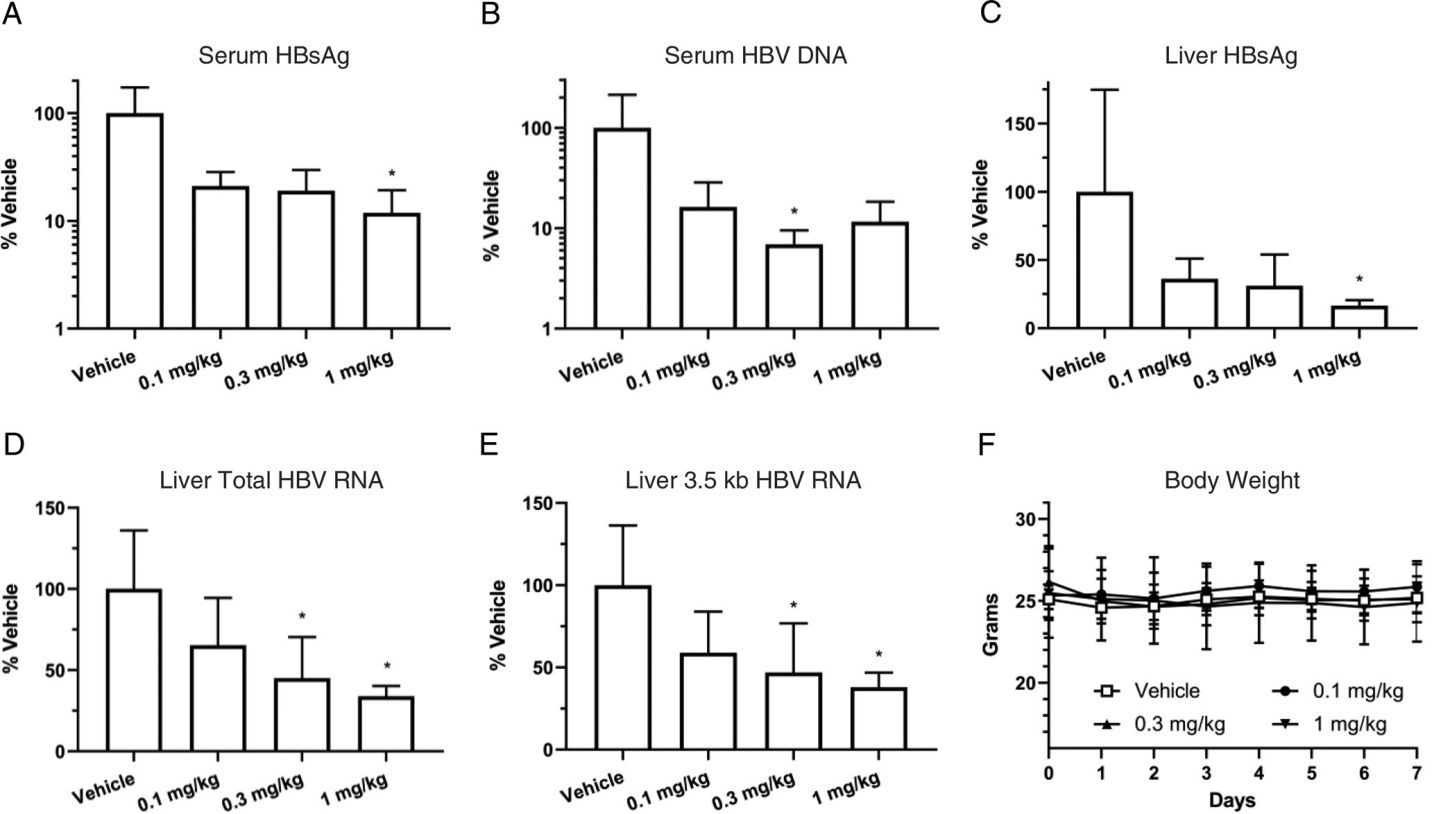
Antiviral activity of AB-452 in an AAV-HBV-transduced mouse model. AAV-HBV-transduced animals received AB-452 at 0.1, 0.3, or 1 mg/kg or vehicle orally twice daily for 7 days. Effect of AB-452 on the production of serum HBsAg (A), serum HBV DNA (B), intrahepatic HBsAg (C), intrahepatic total HBV RNA (D), and intrahepatic 3.5 kb HBV pgRNA (E) on day 7 posttreatment. (F) Effect of AB-452 on body weight through the 7-day treatment. Data represent group mean (*n *= 5) ± SD. Statistically significant difference (*P* < 0.05) from vehicle control was determined using one-way analysis of variance (ANOVA) (Dunn’s multiple-comparison test) and is denoted by an asterisk.

An adeno-associated virus (AAV)-HBV-transduced mouse model was used to assess the anti-HBV effect of AB-452 *in vivo* (Fig. 2). Compared to the vehicle control, oral administration of AB-452 for 7 days at 0.1, 0.3, and 1 mg/kg twice daily resulted in mean 0.68-, 0.72-, and 0.93-log_10_ reduction of serum HBsAg (Fig. 2A) and mean 0.79-, 1.16-, and 0.94-log_10_ reduction of serum HBV DNA (Fig. 2B), respectively. Inhibition of circulating HBV markers at 0.1-, 0.3-, and 1-mg/kg doses was found to be correlated with dose-dependent reductions of viral products in the liver: intrahepatic HBsAg levels were reduced by 64, 69, and 83% (Fig. 2C), intrahepatic total HBV RNA levels were reduced by 35, 55, and 66%, and intrahepatic pgRNA levels were reduced by 43, 55, and 63% (Fig. 2D and E), respectively. AB-452 treatments were well tolerated, with no significant change or reduction in body weight in mice throughout the course of the compound treatment compared to those receiving the vehicle control (Fig. 2F). The *in vitro* observation that AB-452 suppressed intracellular HBV RNA was therefore translatable to the *in vivo* AAV-HBV-transduced mouse model when treated with AB-452.

### AB-452 promotes HBV RNA degradation through inhibiting PAPD5/7 and blocking guanosine incorporation within HBV poly(A) tails.

**FIG 3:**
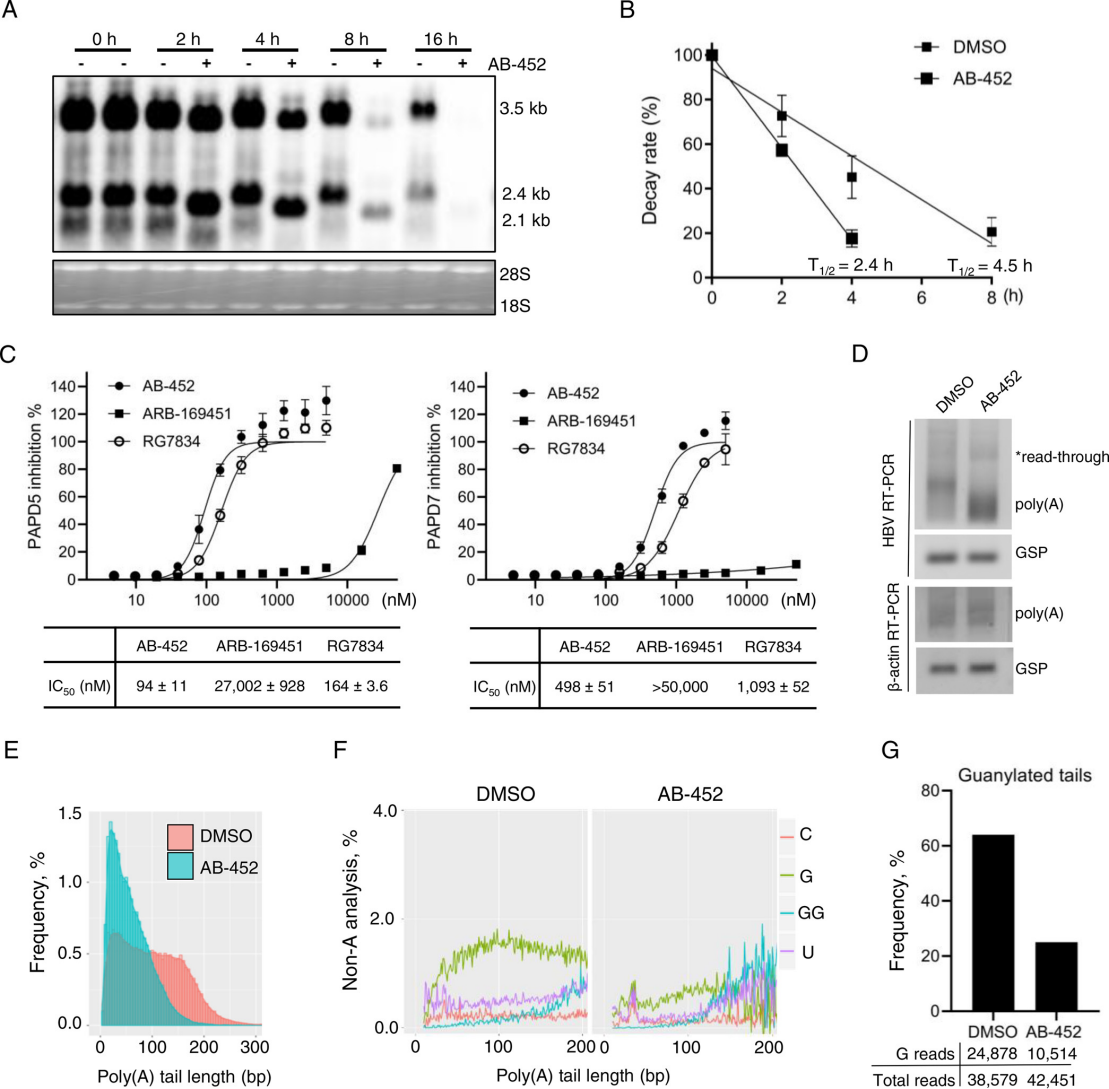
AB-452 promotes HBV RNA degradation through inhibiting PAPD5 and PAPD7 enzymatic activities and blockage of guanosine incorporation into viral RNA poly(A) tails. HepAD38 cells were cultured in the absence of Tet to promote HBV transcription and in the presence of the capsid inhibitor GLS-4 to prevent pgRNA encapsidation for 6 days. On day 7, Tet was added back with media containing either DMSO or AB-452 (70 nM), and cells were harvested either before treatment (time zero h) or at 2, 4, 8, and 16 h posttreatment. (A) HBV mRNA was analyzed by Northern blotting, with ribosomal RNAs as loading control. The positions of HBV pgRNA (3.5 kb) and subgenomic RNAs (2.4 kb and 2.1 kb) are indicated. (B) Decay rate of HBV pgRNA in the presence or absence of AB-452, with calculated *t*_1/2_ labeled for each treatment (*n *= 2). (C) Effect of AB-452, RG7834, and AB-169451 on the enzymatic activity of PAPD5 and PAPD7. Half-maximal inhibition (IC_50_) for the three compounds was determined based on the dose-response curves and reported in the table below each figure. Mean values (± standard derivations) are presented from duplicate experiments. (D to G) Analysis of HBV poly(A) tails from HepAD38 cells treated with or without AB-452 (70 nM). (D) Cells were incubated with AB-452 for 4 h prior to isolation of intracellular RNA. Total RNA was tagged with a poly(G/I) tail at the 3′ end and reverse transcribed by poly(G/I)-specific primer. Both HBV and β-actin mRNA poly(A) tails were specifically amplified using one gene-specific primer and the universal primer that anneals to the G/I tail. Gene-specific PCR (GSP) was used as loading control. The poly(A) tail length of β-actin mRNA served as the negative control. (E) HBV RNA poly(A) tails were subjected to next-generation sequencing for tail lengths analysis. (F) Frequency of non-A modifications (C, cytidylation; G/GG, guanylation; U, uridylation) was analyzed within the HBV poly(A) tails. Guanosines were often clustered; tandem GG analysis was made to reflect this observation. (G) Determination of G nucleotide frequency located within the HBV poly(A) tails. The numbers of guanylated tail reads and total tail reads obtained from NGS were indicated under each sample. The frequency of guanylated tails of viral mRNAs was calculated for poly(A) tail length of ≥10 nt.

To investigate the molecular mechanism of how AB-452 inhibits HBV RNA, studies were performed using HepAD38 cells in which HBV transcription is under tetracycline (Tet) regulation. Tet was first removed to induce transcription and accumulation of viral RNAs, and the capsid inhibitor GLS-4 was added to prevent pgRNA encapsidation. Six days later, Tet was added back to shut down further transcription, and cells were treated with both GLS-4 and AB-452 for an additional 16 h. The effect of AB-452 on HBV transcripts was evaluated by collecting cells at 0, 2, 4, 8, and 16 h posttreatment, and decay of the transcribed HBV RNA was monitored by Northern blot analysis. In the absence of AB-452, HBV RNA levels reduced over time due to natural decay (Fig. 3A). In the presence of AB-452, both pgRNA and sRNA exhibited faster migration starting at 2 h posttreatment, and their levels were significantly reduced at 8 and 16 h posttreatment (Fig. 3A). Determination of the pgRNA half-lives (*t*_1/2_) showed that AB-452 treatment reduced the *t*_1/2_ values from 4.5 h to 2.4 h compared to those from untreated cells (Fig. 3B).

Destabilization of HBV RNA by RG7834 was reported to be mediated through inhibiting the PAPD5 and PAPD7 proteins (32, 37). We determined the effect of AB-452 on the enzymatic activity of recombinant human PAPD5 and PAPD7 using an ATP depletion biochemical assay (Fig. 3C). Results showed that AB-452 efficiently inhibited PAPD5 with a half-maximal inhibition concentration (IC_50_) of 94 nM (Fig. 3C). RG7834 also inhibited PAPD5, although the potency (IC_50_, 167 nM) was lower than previously reported (IC_50_, 1.3 nM) (32). We speculate that the reduction in the inhibitory effect against the recombinant enzymes could be due to the truncated PAPD5 form that was used in the current study. Both AB-452 and RG7834 inhibited PAPD7, with IC_50_ values of 498 nM and 1,093 nM, respectively. In contrast, the enantiomer ARB-169451 was unable to effectively inhibit PAPD5 (IC_50_, 27,000 nM) or PAPD7 (IC_50_ > 50,000 nM) (Fig. 3C).

RNA metabolism in most eukaryotic mRNAs employs the 3′ deadenylation pathway in which poly(A) tail shortening is often observed prior to mRNA degradation (38–40). We therefore determined the HBV poly(A) tail length and composition from HepAD38 cells in the presence or absence of AB-452. To amplify the HBV poly(A) tail, G/I (guanosine and inosine nucleotides) tailing was added to the 3′ ends of mRNA transcripts, and the newly added G/I tails were used as the priming sites to synthesize the cDNA that would be used for amplification of HBV poly(A) tails. The lengths and compositions of the amplicons containing the HBV RNA poly(A) tails were analyzed by next-generation sequencing (PacBio Sequel sequencing platform). Results showed that majority of the HBV poly(A) tails from untreated samples ranged between 50 to 200 nucleotides in length, with an average length of around 100 nucleotides. In contrast, AB-452 treatment reduced the HBV RNA poly(A) tail length by almost 50%, to an average of 58 nucleotides (Fig. 3D and E). The poly(A) tails amplified from β-actin cDNAs served as the negative control, which was not affected by the treatment (Fig. 3D).

It was recently reported that PAPD5/7 extended HBV mRNA poly(A) tails with intermittent guanosine (G), and the incorporation of G could shield them from rapid deadenylation by cellular deadenylases (33). Since AB-452 inhibited PAPD5/7 enzymatic activities and shortened poly(A) tail lengths, we therefore hypothesized that the G content within the HBV poly(A) tails would be affected by AB-452 treatment. Quantification of the nonadenosine nucleosides within the HBV poly(A) tails indeed revealed that the frequency of G was significantly reduced in the presence of AB-452 (Fig. 3F). The fraction of poly(A) tails containing internal G was reduced from 64% to 25% in the presence of AB-452 compared to those from untreated HepAD38 cells (Fig. 3G). Taken together, the data indicate that inhibition of PAPD5/7 by AB-452 led to blockage of G incorporation and shortening of the poly(A) tail.

### SLα within the PRE sequence is required for HBV RNA integrity and AB-452 susceptibility.

**FIG 4:**
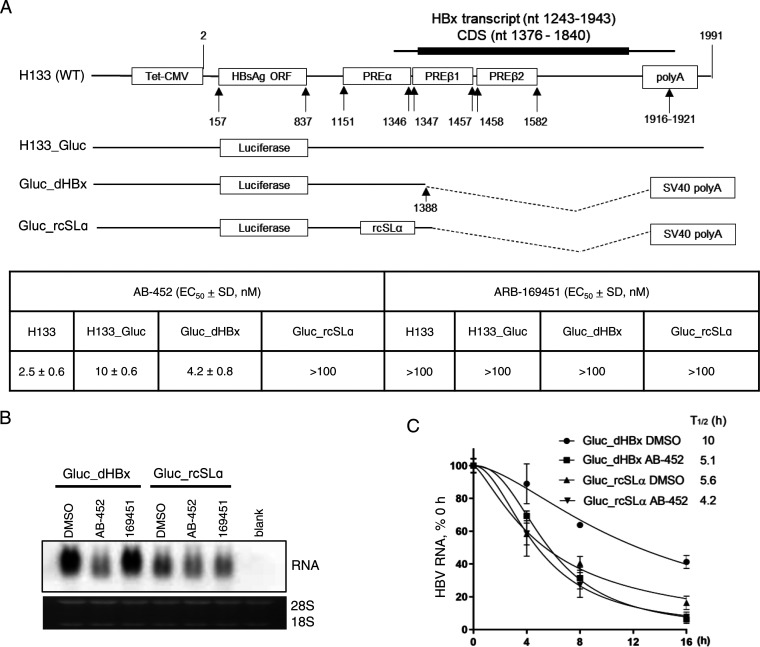
SLα sequence within the HBV PREα subelement is essential for RNA stability and AB-452 activity. Evaluation of AB-452 against HBsAg and *Gaussia* luciferase (Gluc)-encoded plasmids containing either wild-type HBV PREα (H133, H133_Gluc, or Gluc_dHBx) or SLα inversion-derived mutant (Gluc_rcSLα). (A) Schematic representation of H133 (wild-type) and the Gluc-derived constructs. Huh-7 cells were transfected with each of these plasmids, and susceptibility to AB-452 was evaluated by monitoring HBsAg or Gluc activity. The inactive enantiomer ARB-169451 was included as a negative control. Mean values (± standard derivations) are presented from triplicate experiments. (B) The HBx deletion variants containing either the WT SLα (Gluc_dHBx) or the inverted SLα (Gluc_rcSLα) sequence were transfected into Huh-7 cells, which were treated with DMSO, AB-452 (100 nM), or ARB-169451 (100 nM) for 5 days. Effect of AB-452 against HBV RNA was analyzed by Northern blotting with ribosomal RNAs as loading control. (C) Kinetics of HBV RNA degradation in cells transfected with Gluc_dHBx or Gluc_rcSLα. Transcription proceeded for 2 days prior to the addition of tetracycline with or without AB-452 (100 nM). Cells were harvested before treatment (time 0 h) and at 4, 8, and 16 h posttreatment. HBV RNA decays were monitored by qRT-PCR assay, with calculated decay *t*_1/2_ labeled under each treatment (*n *= 3). Data and error bars represent mean percentage of HBV RNA and standard deviations relative to time zero of each condition from at least three independent experiments.

We and others have determined that reduction of HBV RNA by RG7834 is dependent on the HBV PRE (32, 36), which partially overlapped with the HBx coding region. To further define the involvement of the subelements within PRE on HBV RNA stability and AB-452 susceptibility, the following several reporter plasmids were constructed (Fig. 4A): (i) H133 is the wild-type construct supporting the expression of 2.1 kb HBV sRNA expression, (ii) H133_Gluc is derived from H133 but with the HBsAg coding sequence replaced with *Gaussia* luciferase, (iii) Gluc_dHBx is derived from H133_Gluc but with most of the HBx coding sequence deleted (nucleotides 1389 to 1991) and the HBV poly(A) replaced with the SV40 poly(A) signal, and (iv) Gluc_rcSLα is derived from Gluc_dHBx with an inverted SLα sequence.

AB-452, but not its enantiomer ARB-1694151, inhibited both HBsAg and Gluc expression in cells transfected with H133 (EC_50_, 2.5 nM), H133_Gluc (EC_50_, 10.0 nM), or the Gluc_dHBx construct (EC_50_, 4.2 nM) (Fig. 4A). These data indicate that AB-452 antiviral activity was not dependent on the HBsAg sequence, HBx sequence, or the HBV poly(A) signal sequence. On the other hand, inverting the SLα sequence (Gluc_rcSLα) abolished sensitivity to AB-452 (EC_50_ > 100 nM) (Fig. 4A and B). Interestingly, we observed that the transcribed RNA from the Gluc_rcSLα-transfected cells showed reduction in RNA levels and appeared smaller in size than the RNA from cells transfected with the Gluc_dHBx plasmid, with or without AB-452 treatment (Fig. 4B). The rates of HBV RNA decay revealed that AB-452 treatment reduced Gluc_dHBx RNA half-lives (*t*_1/2_) from 10 h to 5.1 h compared to dimethyl sulfoxide (DMSO)-treated cells (Fig. 4C). In contrast, the Gluc_rcSLα RNA was unstable (*t*_1/2_, 5.6 h), and its *t*_1/2_ was only slightly reduced by AB-452 (*t*_1/2_, 4.2 h) (Fig. 4C).

**FIG 5:**
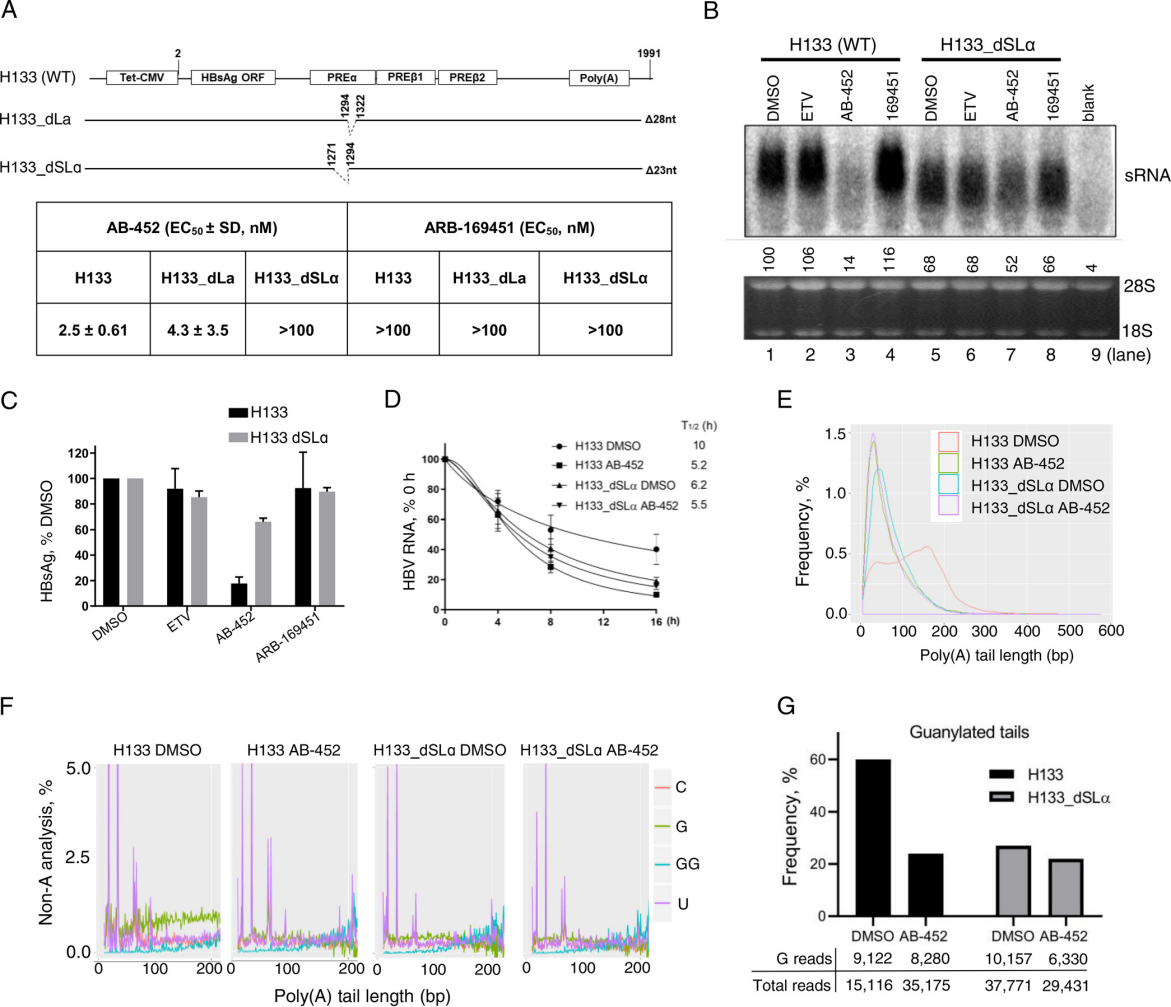
SLα determines AB-452 sensitivity and HBV RNA poly(A) tail integrity. Huh-7 cells were transfected with H133 (wild-type), H133_dLa, or H133_dSLα plasmids and treated with AB-452 for 5 days. (A) Schematic representation of H133 (wild-type), H133_dSLα (SLα deleted, nt 1294 to 1322), and H133_dLα mutants (La binding site deleted, nt 1271 to 1294). The activities of AB-452 and ARB-169451 against HBsAg production were determined and EC_50_ values summarized. Mean values (± standard derivations) are determined from triplicate experiments. (B and C) H133 and H133_dSLα were transfected into Huh-7 cells and treated with ETV (1 μM), AB-452 (0.1 μM), or ARB-169451 (0.1 μM) for 5 days. Intracellular HBV sRNA and secreted HBsAg were analyzed by Northern blotting or ELISA, respectively. Levels of HBV RNA and HBsAg were normalized to those from cells treated with DMSO. Data represent average values ± standard deviations from at least three independent experiments. (D) Kinetics of HBV RNA degradation in Huh-7 cells transfected with H133 and H133_dSLα plasmids. Transcription proceeded for 2 days prior to the addition of tetracycline with or without AB-452 (100 nM). Cells were harvested before treatment (time, 0 h) and at 4, 8, and 16 h posttreatment. HBV RNA decays were monitored by qRT-PCR assay, with calculated decay *t*_1/2_ labeled (*n *= 3). (E) HBV RNA poly(A) tails were sequenced and analyzed for frequency of tail lengths from cells transfected with H133 or H133_dSLα treated with or without AB-452 (100 nM). (F) Frequency of non-A modifications (G, guanylation; U, uridylation; C, cytidylation) within the poly(A) tail of HBV mRNAs were analyzed. Tandem GG analysis was performed to analyze clustered guanosines. (G) The frequency of guanylated tails of viral mRNAs was calculated with a poly(A) tail length of ≥10 nt. The numbers of guanylated tail reads and total tail reads obtained from the NGS were indicated under each sample.

In addition to SLα, HBV PREα contains another *cis*-acting element known as La protein binding element; these two *cis*-acting elements were included in a 109-nucleotide sequence that was critical for RG7834 sensitivity (36). The requirement of these two elements was studied by generating two additional H133-derived constructs, H133_dSLα and H133_dLa, in which the SLα sequence and the La element were deleted, respectively. AB-452 inhibited HBsAg production in H133_dLa-transfected cells with similar efficiencies as the wild-type H133 construct (EC_50_s, 4.3 and 2.5 nM, respectively), indicating that the La protein binding element was not essential for susceptibility to AB-452. Like the results observed in the Gluc_rcSLα transfection, AB-452 was inactive against the H133_dSLα (EC_50_ > 100 nM) (Fig. 5A). Deleting the SLα sequence also led to the shortening and reduction of sRNA level (Fig. 5B), as well as reduced transcript *t*_1/2_ (Fig. 5D). Cells transfected with H133_dSLα showed reduced sensitivity to AB-452 compared to the wild-type H133-transfected cells (Fig. 5B and C). The transcript half-life from the H133_dSLα-transfected cells treated with AB-452 was only slightly reduced from 6.2 h to 5.5 h compared to those treated with DMSO (Fig. 5D).

Next-generation sequencing (NGS) analysis of the sRNA poly(A) tails showed that AB-452 reduced the average poly(A) tail length of H133 transcripts from 124 to 64 nucleotides (Fig. 5E). The poly(A) tails from the H133_dSLα transcripts were 62 and 71 nucleotides with and without AB-452 treatment, respectively (Fig. 5E). In terms of the poly(A) tail composition, the guanylation frequency was highest in cells transfected with the wild-type PRE (H133), and the overall guanylation frequency was reduced from about 60% to 24% in the presence of AB-452 (Fig. 5F and G). In contrast, the guanylation frequency in the H133_dSLα transcripts already appeared low (22% to 27%) with and without AB-452 treatment (Fig. 5F and G). Taken together, these data provide first-line evidence demonstrating that the SLα sequence serves to stabilize the viral transcripts through maintaining the poly(A) tail lengths and mixed-nucleotides composition.

### PAPD5 and PAPD7 determine HBV RNA integrity and stability.

**FIG 6:**
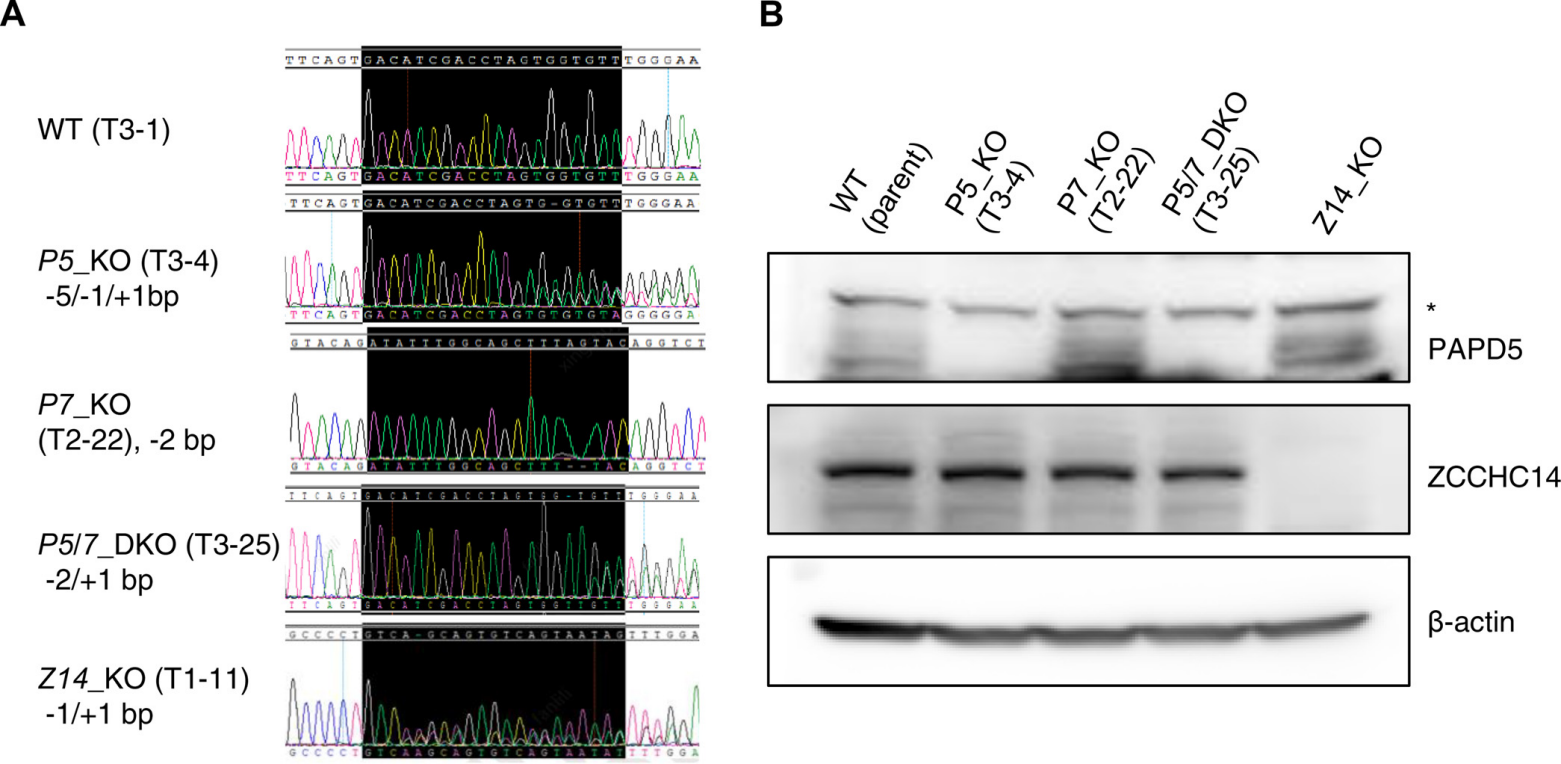
Confirmation of the *PAPD5*, *PAPD7*, and *ZCCHC14* CRISPR-Cas9-mediated knockouts in the representative clones. (A) Insertion-deletion mutations (indels) are annotated with the bps of insertion (+) or deletion (−) on alleles, in which “/” is used to separate indels among different alleles. The regions targeted by gRNAs are highlighted in black. Indels are detected by sequencing trace analysis with CAT tool (CRISPR analysis tool). (B) PAPD5 and ZCCHC14 were detected with the indicated antibodies in the Western blots. Asterisk indicates a cross-reacting band.

To understand the individual role of PAPD5 and PAPD7 in regulating HBV RNA stability and poly(A) tail integrity, knockout (KO) cell lines with deletion of *PAPD5* (*P5*_KO), *PAPD7* (*P7*_KO), or both *PAPD5/7* (double knockout, *P5/7*_DKO) were isolated using CRISPR-Cas9 gene editing and HepG2-NTCP cells. It was reported that ZCCHC14 (Z14), which interacts with PAPD5/7 and the HBV PRE, plays an important role in maintaining HBV RNA integrity and stability (33). *Z14* KO cell lines (*Z14*_KO) were generated to assess the involvement of Z14 in regulating HBV RNA. In addition to the parental wild-type (WT) HepG2-NTCP cell line, two additional WT cell clones (T3-4 and T2-14) were included as clonal controls. The full-allelic KO genotype for all the individual cell clones was confirmed by DNA sequencing (Fig. 6A), and *PAPD5* and *Z14* knockout were also confirmed at the protein level (Fig. 6B). PAPD7 protein expression could not be evaluated by Western blotting due to the lack of an efficient PAPD7-specific antibody, but the PAPD7 KO genotype was confirmed by DNA sequencing (Fig. 6A).

**FIG 7:**
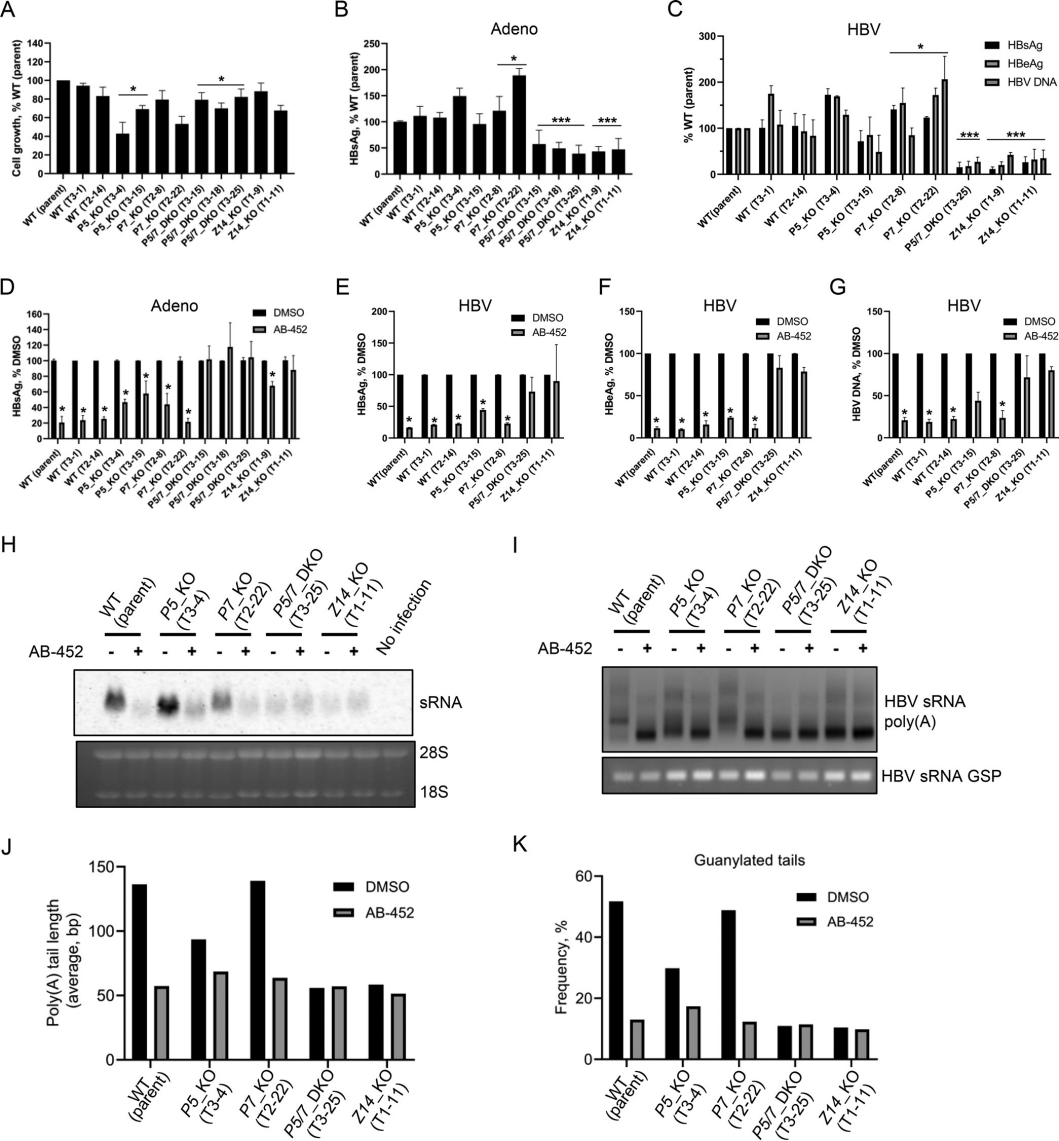
Knockout of *PAPD5/7* and *ZCCHC14* destabilizes and desensitizes HBV RNA to AB-452. *PAPD5*, *PAPD7*, *ZCCHC14*, or both *PAPD5* and *PAPD7* were knocked out in HepG2-NTCP cells by CRISPR-Cas9 gene editing. (A) Cell proliferation of *PAPD5*, *PAPD7*, and *Z14* KO or WT clones was analyzed. Percentage of cell growth relative to the WT parent HepG2-NTCP cells was determined for each tested clone. (B) Adenoviruses carrying HBsAg coding sequence were used to transduce either WT, *PAPD5*, *PAPD7*, or *ZCCHC14* KO cell clones; extracellular HBsAg was measured on day 5 postransduction. Percentage of HBsAg relative to the WT parent HepG2-NTCP cells was determined. (C) HepG2-NTCP cells were infected with HBV inoculum. HBsAg, HBeAg, and HBV DNA were measured on day 9 postinfection in the KO clones and normalized to the WT parent cells. (D) AB-452 activity of HBsAg inhibition was evaluated in the *PAPD5/7* single- or double-KO and *ZCCHC14* KO clones infected with adenoviruses. (E to G) AB-452 antiviral activity was evaluated in HBV-infected HepG2-NTCP clones. (H) HBV sRNA was analyzed by Northern blotting in the *PAPD5/7* single- or double-KO and *ZCCHC14* KO cell clones treated with and without AB-452 for 5 days. (I) HBV mRNA poly(A) tails were amplified, and the obtained amplicons were resolved in a 2% agarose gel. Gene specific PCR (GSP) of HBV RNA was used as loading control. (J and K) HBV sRNA poly(A) tails were sequenced for the analysis of tail lengths (J) and guanylation incorporation frequency (K). The mean values and standard derivations were plotted at least from duplicate experiments for the panels A to G. *, *P < *0.05; **, *P < *0.01; ***, *P < *0.001 compared with three WT cell lines or DMSO-treated cells. *P* values were calculated by unpaired two-tailed *t* test.

Overall, cell proliferation analysis suggests that PAPD5, PAPD7, PAPD5/7, and Z14 were not critical for cell survival (Fig. 7A). The effect of knocking out *PAPD5/7* and *Z14* on viral protein production and HBV replication was examined by using two independent systems, adenovirus-encoded HBsAg transduction and HBV infection (Fig. 7B and C). In the adenovirus transduction studies, a single KO of *PAPD5* or *PAPD7* did not reduce HBsAg production compared to the WT cell clones. HBsAg expression in the *P5/7*_DKO and *Z14*_KO clones was about 50% lower than that of the WT or *PAPD5/7* single KO clones in the 5-day culture (Fig. 7B). In the HBV infection studies, the levels of viral proteins and HBV DNA were much lower in the *P5/7*_DKO and *Z14*_KO clones than the WT or *PAPD5/7* single-KO clones in the 9-day culture (Fig. 7C).

We next examined the impact of deleting *PAPD5*, *PAPD7*, *Z14*, or both *PAPD5/7* on compound sensitivity. In adenovirus-transduced cells, AB-452 inhibited HBsAg production from WT (EC_50_, 9.0 nM) and *P7*_KO cell clones (EC_50_s, 7.1 and 10.0 nM) with similar EC_50_ values. However, AB-452 was about 6- to 8-fold less active against the *P5*_KO cell clones (EC_50_s, 56.0 and 71.6 nM) (Table 4). Susceptibility to AB-452 was also evaluated using HBV-infected HepG2-NTCP cells: results showed that AB-452 inhibited WT (EC_50_, 2.7 nM) and *P7*_KO (EC_50_s, 3.2 and 3.8 nM) cells with similar efficiencies but was again about 10-fold less active against the *P5*_KO cells (EC_50_s, 37.7 and 39.3 nM) (Table 4). A similar trend was also observed with RG7834, suggesting this differentiated antiviral activity was not AB-452 specific. The antiviral data are consistent with the finding that AB-452 and RG7834 were more efficient against PAPD5 than PAPD7 in the enzymatic assays (Fig. 3C). Among the *P5/7*_DKO and *Z14*_KO cell lines, AB-452 treatment did not show further inhibition than the untreated controls (Fig. 7D to G).

**TABLE 4:** Anti-HBV effect of AB-452 and RG7834 in *PAPD5* or *PAPD7* KO cell lines^*a*^

Cell line	Compound	Adeno infection		HBV infection	
		EC_50_ (nM)	FC vs WT	EC_50_ (nM)	FC vs WT
WT (parent)	AB-452	9.0 ± 4.6	1	2.7 ± 1.8	1
	RG7834	11.8 ± 9.6	1		
*P5*_KO (T3-4)	AB-452	71.6 ± 31.2	8.0	37.7 ± 8.3	14.0
	RG7834	126 ± 74.7	10.7		
*P5*_KO (T3-15)	AB-452	56.0 ± 34.0	6.3	39.3 ± 3.2	14.6
	RG7834	85.7 ± 59.8	7.3		
*P7*_KO (T2-8)	AB-452	10.0 ± 1.0	1.1	3.8 ± 1.5	1.4
	RG7834	23.7 ± 15.2	2.0		
*P7*_KO (T2-22)	AB-452	7.1 ± 1.3	0.8	3.2 ± 1.9	1.2
	RG7834	10.8 ± 2.1	0.9		

a Mean EC_50_ ± standard deviations were determined from three independent experiments. FC, fold change.

Interestingly, while there was no appreciative reduction of HBV protein and DNA production observed from the *P5*_KO cells (Fig. 7B and C), we observed that the sRNA migrated faster than that from the WT and *P7*_KO cells (Fig. 7H and I). To determine the exact sRNA length, the sRNA from WT and the various KO cells was further characterized by NGS analysis. Results revealed that knocking out *PAPD5* alone, but not *PAPD7*, reduced both the poly(A) tail lengths (from >136 bp to 94 bp) and guanylation frequency of sRNA (from ∼50% to 30%) compared to the WT cells (Fig. 7J and K). AB-452 treatment led to reduction of poly(A) tail length (from >136 bp to ∼60 bp) and guanylation (from ∼50% to ∼10%) in both WT and *P7*_KO cells. The *P5*_KO cells appeared less sensitive to AB-452 in their shortening of poly(A) tail lengths and guanosine incorporation. Cells with *Z14*_KO and *PAPD5/7*_DKO already showed drastically reduced levels of sRNA, poly(A) tail lengths (51 to 58 bp), and guanosine incorporation (∼10%), with and without AB-452 treatment. Taken together, these results suggest that of the two noncanonical poly(A) polymerases, PAPD5 appeared to play a major role in determining viral poly(A) tail integrity, guanosine incorporation, and AB-452 sensitivity.

## DISCUSSION

Current therapies for chronic hepatitis B patients rarely achieve functional cure, which is characterized as sustained loss of HBsAg with or without HBsAg antibody seroconversion (41). The discovery of RG7834 has raised significant interest, as this class of small-molecule inhibitors has the potential to reduce both HBV RNA and viral proteins, which are distinct from direct-acting antivirals targeting the HBV polymerase and capsid proteins (34, 36, 37). AB-452 is an analog of RG7834 with a similarly broad antiviral effect against multiple HBV replication intermediates. It has been appreciated that integrated HBV DNA is a major source of HBsAg expression in HBeAg-negative patients (42). Our data indicate that AB-452 can reduce HBsAg produced from cccDNA in HBV-infected cells as well as from integrated HBV DNA in patient-derived hepatocellular carcinoma cells (Table 1). Furthermore, oral administration of AB-452 substantially reduced HBV DNA, HBsAg, HBeAg, and intrahepatic HBV RNA from AAV-HBV-infected mice (Fig. 2). Our studies here provide insights into the mode of action for AB-452 and further characterize the RNA stabilization mechanisms utilized by the virus. Our results demonstrate that the *cis*-acting SLα viral sequence and the transacting host factors PAPD5 and PAPD7 coordinate to protect viral RNA. Interference of such viral-host interactions through small-molecule compounds treatment or genetic mutations led to destabilization of viral transcripts and reduction of HBsAg.

The requirement of PAPD5/7 and ZCCHC14 to form a complex with HBV RNA through the PRE element for stabilizing HBV RNA has been described (32, 33). Since the ZCCHC14/PAPD5/7 complex is recruited onto the SLα sequence, it is conceivable that mutating the SLα sequence may disrupt the binding of the ZCCHC14/PAPD5/7 complex and consequently affect HBV RNA stability. Here, our studies provided the genetic evidence that an intact SLα sequence is indeed critical for maintaining HBV poly(A) tail integrity and stability, as inverting or deleting this sequence both destabilize HBV RNA. Notably, the phenotype of the SLα deletion and inversion mutants resembled the antiviral effect of AB-452: cells treated with AB-452 display the phenotypes of HBV poly(A) tail shortening, reduced guanosine incorporation, and HBV RNA degradation.

Initial studies suggest that PAPD5 and PAPD7 may provide redundant if not identical role(s) in protecting HBV RNA stability (32, 33, 37, 43). However, our results from the *P5*_KO and *P7*_KO cell lines would argue that PAPD5 and PAPD7 may serve two lines of protection in maintaining the stability of HBV RNA. *P5*_KO, but not *P7*_KO, impaired poly(A) tail integrity. Moreover, the phenotypic measurements we monitored so far indicate that the *P7*_KO cells were similar to WT cells, further supporting that PAPD5 expression alone could support viral RNA integrity and stabilization (Fig. 7). These data suggest that PAPD7 did not actively contribute to HBV RNA protection in the presence of PAPD5 but, instead, served as a second line of protection by moderately extending the HBV poly(A) tail when PAPD5 was depleted (Fig. 7I to K). Results from the enzymatic assays show that PAPD5 was more robust than PAPD7 in the extension of poly(A) tails (data not shown), supporting our argument that PAPD5 would be the major host factor in protecting HBV RNA. Immune precipitation experiments conducted by two independent research groups indicated that both PAPD5 and PAPD7 were bound to HBV mRNA, with PAPD7 at a lower level compared to PAPD5 (33, 43). Further studies would be required to clarify the role of PAPD7 in HBV RNA metabolism in WT cells.

Another noteworthy observation from this study is that the two HBV RNA destabilizers, AB-452 and RG7834, displayed different inhibitory efficiencies against PAPD5 and PAPD7. Both compounds were 5- to 7-fold less efficient against the enzymatic activities of PAPD7 than PAPD5, which was, in turn, consistent with the results from cell-based studies in which AB-452 and RG7834 displayed a 6- to 10-fold reduction in activities against HBsAg production in the *P5*_KO cells (in which PAPD7 is present) compared to those from the WT and *P7*_KO cells (in which PAPD5 is present). These data suggest that it may not be critical to completely inhibit PAPD7 to achieve HBV RNA destabilization. These data, together with the genetic studies, support the hypothesis that PAPD5 could be more essential than PAPD7 in stabilizing HBV RNA. Our results further suggest that developing PAPD5-selective inhibitors of HBV replication could be pharmacologically feasible.

**FIG 8:**
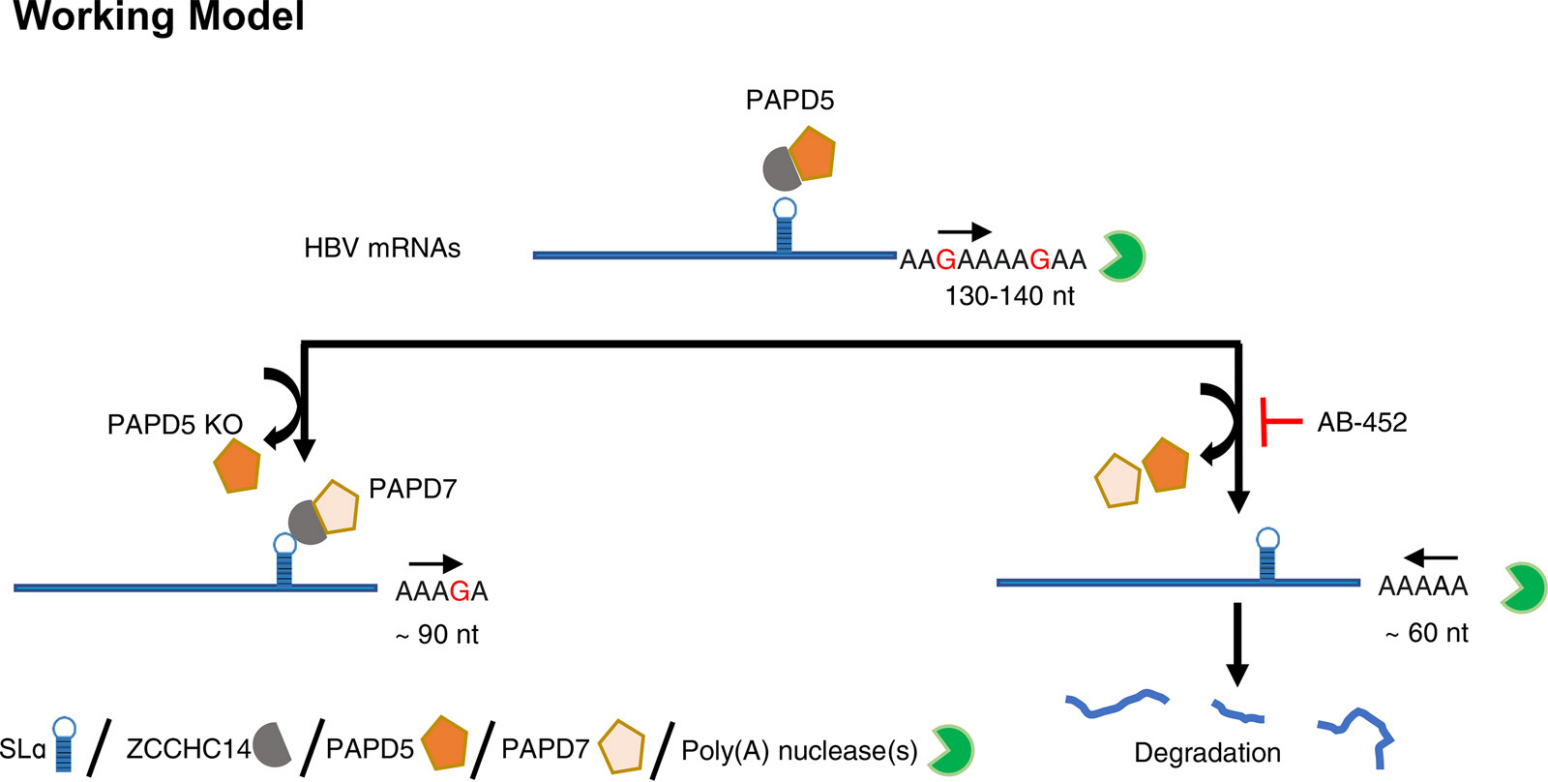
Proposed model illustrating the interplay between HBV *cis* element SLα and the host factors PAPD5 and PAPD7 in maintaining HBV RNA integrity and stability.

Here, we propose a working model of the interplay between HBV transcripts and the cellular ZCCHC14/PAPD5/7 RNA metabolism machineries (Fig. 8). Maintenance of HBV RNA stability is a dynamic process regulated by canonical and noncanonical poly(A) polymerases and deadenylases. PAPD5 could form a complex with ZCCHC14, which directs the noncanonical polymerase onto the viral transcripts through the SLα within the HBV PRE sequence. Assembly of the ZCCHC14/PAPD5 onto SLα within the HBV PRE sequence facilitates the addition of G while extending the poly(A) tail. This guanylation process may stall the cellular poly(A) exonuclease and terminate further deadenylation, thus protecting the RNA from degradation. When PAPD5 is depleted, ZCCHC14/PAPD7 complex may bind to HBV RNA and protects its degradation; however, PAPD7 is less effective for poly(A) extension and guanylation incorporation. When HBV is challenged by HBV RNA destabilizers such as AB-452 or PRE mutations, viral RNA integrity and stability are disrupted due to either the inhibition of PAPD5/7 enzymatic activities or disarraying of the ZCCHC14/PAPD5/7 complex from interacting with the SLα sequence, respectively.

## MATERIALS AND METHODS

### Cell lines and culture.

HepG2.2.15, HepAD38, and PLC/PRF/5 cells were cultured in Dulbecco’s modified Eagle medium (DMEM)/F12 medium (Corning, NY, USA), supplemented with 10% fetal bovine serum (Gemini, CA, USA), 100 U/ml penicillin, and 100 μg/ml streptomycin. Huh-7 cells (Creative Bioarray, NY, USA) were cultured in RPMI 1640 medium (Basel, Switzerland) containing 10% fetal bovine serum, 100 U/ml penicillin, and 100 μg/ml streptomycin. HepG2-hNTCP-C4 cells were cultured in DMEM medium (Gibco, MA, USA) containing 10% fetal bovine serum and 10 mM HEPES (Gibco).

### Antiviral studies.

HepG2.2.15 (30,000 cells/well) were plated in 96-well collagen-treated plates and incubated at 37°C. Compounds were half-log serially diluted and added in duplicate to 96-well microtiter plates. The plates were incubated for a total duration of 6 days, after which the culture medium was removed for the HBsAg and HBeAg CLIA assays (AutoBio Diagnostics Co., China) performed according to the manufacturer’s instructions. Secreted HBV DNA was extracted (Realtime Ready cell lysis kit; Roche, Mannheim, Germany) and quantified in a qPCR assay (LightCycler 480 SYBR green I master mix; Roche) with the 5′-GGCTTTCGGAAAATTCCTATG-3′ (sense) and 5′-AGCCCTACGAACCACTGAAC-3′ (antisense) primers using the PCR conditions of denaturing at 95°C for 5 min followed by 40 cycles of amplification at 95°C for 15 s and 60°C for 30 s. Antiviral selectivity studies against a panel of different DNA and RNA viruses were performed at ImQuest BioSicences (Frederick, MD, USA). Briefly, test compounds were evaluated using 6 doses, including the highest concentration of 30 μM and five serial half-logarithmic dilutions in triplicate for the antiviral assays.

### Infection of HepG2-hNTCP-C4 cells and PHHs.

HepG2-hNTCP-C4 cells were seeded into collagen-coated 10-cm dishes at a density of 8.6 × 10^6^ cells per dish and cultured in 10 ml complete DMEM medium containing 2% dimethyl sulfoxide (DMSO). One day later, the cells were infected with HBV at 100 to 250 genome equivalent (GE)/cell in DMEM containing 4% polyethylene glycol 8000 (PEG 8000). The inoculums were removed 24 h later, and the infected cells were washed 4 times with phosphate-buffered saline (PBS) and seeded in 96-well plates at a density of 5 × 10^4^/well following trypsinization. Serial dilutions of compounds were added to the plates and refreshed at days 4, 8, and 11 postinfection. The supernatants were harvested for HBsAg and HBeAg analysis (enzyme-linked immunosorbent assays [ELISAs]; International Immuno-Diagnostics, CA, USA). HBV DNA was extracted from cell lysates per the manufacturer’s instructions (Qiagen DNeasy 96 blood and tissue kit; Qiagen, Hilden, Germany). HBV DNA was detected by qPCR using primers and probe as follows: 5′-GTC CTC AAY TTG TCC TGG-3′ (sense), TGA GGC ATA GCA GCA GGA-3′ (antisense), and Probe/56-FAM/CTG GAT GTG TCT GCG GCG TTT TAT CAT/36-TAMSp/. For the infection of PHHs, cells were placed in collagen-coated 96-well plates (65,000 cells/well) overnight and then infected with HBV at 250 GE per cell in media containing 4% PEG 8000. The inoculums were removed 24 h later, and compounds were added or replenished on days 4, 7, 9, 11, and 14 postinfection. On day 16, medium was removed, and HBV antigens and DNA and RNA quantification were monitored as described above.

### Detection of intracellular HBV RNA by Northern blotting analysis.

Total cellular RNA was extracted from HepG2.2.15 or HepAD38 cells treated with or without antiviral compounds using TRIzol reagent according to the manufacturer's directions (Thermo Fisher Scientific, Waltham, MA). Northern blotting was performed as described previously (1). Briefly, total RNA was separated in a 1.5% agarose gel and transferred onto an Amersham Hybond-XL membrane (product no. GERPN303S; GE Healthcare, IL, USA). Membranes were probed with an [α-^32^P]UTP (PerkinElmer, CT, USA)-labeled minus strand-specific full-length HBV riboprobe (3.2 kb) transcribed from plasmid pSP65_HBV(+) DNA. Membranes were exposed to a phosphoimager screen, and the hybridization signals were quantified using Image Studio software (Li-Cor Biosciences, NE, USA).

### Detection of intracellular HBV DNA by Southern blotting.

Total intracellular viral core-associated DNA was extracted as described previously (1) and analyzed by Southern blotting hybridization with an [α-^32^P]UTP-labeled full-length HBV riboprobe (3.2 kb) that specifically hybridized to minus strand of viral DNA. Membranes were exposed to a phosphoimager screen, and the hybridization signals were quantified using Image Studio software.

### Detection of intracellular HBcAg, PAPD5, and β-actin by Western blotting.

HepG2.2.15 or HepAD38 cells cultured in a 12-well plate were lysed with 200 μl Laemmli sample buffer (Bio-Rad, PA, USA) supplemented with 2.5% 2-mercaptoethanol (Sigma-Aldrich, MO, USA). Cell lysates were subjected to denaturing gel electrophoresis with 12% Criterion TGX stain-free precast gels and Tris-glycine-SDS running buffer (Bio-Rad). Proteins were transferred from the gel onto a polyvinylidene difluoride (PVDF) membrane Trans-Blot Turbo transfer system (Bio-Rad). Membranes were blocked with 5% nonfat milk in Tris-buffered saline (TBS)-0.1% Tween for 1 h and incubated with the primary antibody overnight at 4°C. After washing with TBS containing 0.1% Tween 20 (TBST), the membrane was incubated with the secondary antibody. Membranes were again washed 3 times with TBST and soaked with 200 μl Clarity Western ECL substrate (Bio-Rad) and imaged with the iBright imaging systems (Thermo Fisher Scientific). The primary antibodies used in the present study include anti-HBc antibody (catalog no. B0586; Dako, United Kingdom), anti-PAPD5 antibody (catalog no. HPA042968; Atlas Antibodies, Bromma, Sweden), and anti-β-actin antibody (catalog no. ab8227; Abcam, Cambridge, United Kingdom).

### Particle gel for viral nucleocapsid and encapsidated HBV DNA analysis.

For intracellular viral nucleocapsid analysis, HepG2.2.15 cells were lysed in buffer containing 10 mM Tris-HCl (pH 7.6), 100 mM NaCl, 1 mM EDTA, and 0.1% NP-40. Cell debris was removed by centrifugation, and the viral particles were fractionated through nondenaturing 1% agarose gels electrophoresis and transferred to a nitrocellulose filter by blotting with TNE buffer (10 mM Tris-HCl [pH 7.6], 150 mM NaCl, and 1 mM EDTA). To detect HBV core antigens, membranes were probed with polyclonal antibody against HBV core protein (catalog no. B0586; Dako, United Kingdom). Bound antibodies were revealed by horseradish peroxidase (HRP)-labeled secondary antibodies (Thermo Fisher Scientific) and visualized with the iBright imaging systems according to the protocol of the manufacturer. For the detection of encapsidated HBV DNA, the DNA-containing particles on the membrane were denatured with a solution containing 0.5 M NaOH and 1.5 M NaCl, and this step was followed by neutralization with a solution containing 1 M Tris-HCl (pH 7.6) and 1.5 M NaCl. HBV DNA was detected by hybridization with an [α-^32^P]UTP-labeled full-length HBV riboprobe (3.2 kb) that specifically hybridized to the minus strand of viral DNA.

### Detection of encapsidated pgRNA.

To detect intracellular encapsidated pgRNA, HepG2.2.15 or HepAD38 cells were lysed with 300 μl of lysis buffer (10 mM Tris-HCl [pH 7.6], 100 mM NaCl, 1 mM EDTA, and 0.1% NP-40) per well. Cell debris and nuclei were removed by centrifugation, and the supernatants were digested with 20 U/ml of micrococcal nuclease (MNase) at 37°C for 30 min. The core particles were precipitated with 35% PEG 8000 dissolved in 1.5 M NaCl on ice for 60 min, isolated by centrifugation, and dissolved in TNE buffer (10 mM Tris-HCl, pH 7.6, 150 mM NaCl, and 1 mM EDTA). Encapsidated pgRNA in core particles was extracted with TRIzol reagent, and pgRNA was quantified in a qPCR assay (SuperScript III Platinum SYBR Green one-step qPCR kit w/ROX; Invitrogen) with the 5′-GGT CCC CTA GAA GAA GAA CTC CCT-3′ (sense) and 5′-CAT TGA GAT TCC CGA GAT TGA GAT-3′ (antisense) primers using the PCR conditions of 50°C for 30-min hold (cDNA synthesis), denaturing at 95°C for 5 min, followed by 40 cycles of amplification at 95°C for 15 s, 60°C for 30 s, and 72°C for 30 s.

### *In vivo* antiviral activity in a mouse model of HBV.

HBV mouse experiments were conducted at Arbutus Biopharma (Burnaby, Canada) in accordance with Canadian Council on Animal Care (CCAC) Guidelines on Good Animal Practices and using protocols approved through the CCAC-certified Institutional Animal Care and Use program. Male C57BL/6J mice, 6 weeks old, were each inoculated with 1E11 genomes of adeno-associated virus (AAV) vector AAV-HBV1.2 containing a 1.2× overlength sequence of HBV genome (genotype D; GenBank accession no. V01460) (2). Mice were administered the AAV2/8-type vector via intravenous tail vein injection. Twenty-eight days after AAV infection, animals were randomized into groups (*n* = 5) based on serum HBsAg concentration. Animals were administered vehicle only or AB-452 at 0.1, 0.3, or 1 mg/kg by oral gavage twice daily for 7 days and terminated 12 h after the last dose. Serum and liver HBsAg concentrations were determined using the Bio-Rad enzyme immunoassay (EIA) GS HBsAg 3.0 kit according to the manufacturer's instructions. Serum HBV DNA concentrations were measured from total extracted DNA using primer/probe sequences described previously (3). Total HBV RNA concentrations in the liver were quantified using a branched DNA assay (QuantiGene 2.0; Thermo Fisher Scientific) with probes targeting the shared 3′ region of HBV transcripts, whereas 3.5 kb HBV RNA concentrations were quantified with probes targeting the unique 5′ region of the pgRNA; in both cases, signal was normalized against mouse glyceraldehyde 3-phosphate dehydrogenase (GAPDH).

### Purification of recombinant PAPD5 and PAPD7 proteins.

Molecular cloning, expression, and purification of the recombinant human PAPD5 and PAPD7 proteins were performed at Xtal BioStructures (MA, USA). PAPD5 (amino acids 186 to 518; GenBank accession no. XM_011523275) and PAPD7 (amino acids 226 to 558; GenBank accession no. NM_006999.6) genes comprising of nucleotidyltransferase and PAP-associated domains were codon optimized, synthesized, and inserted in the expression vector pET-24a(+) for E. coli expression with 10×His-Flag-tobacco etch virus (TEV) tags at the N terminus. The pET-24a(+) constructs were transformed into E. coli-competent cells BL21(DE3). Cells were grown in TB-kanamycin (Kan) medium (1.2% tryptone, 2.4% yeast extract, 0.4% glycerol, 72 mM K_2_HPO_4_, 17 mM KH_2_PO_4_ plus 50 μg kanamycin/ml plus 100 mM sodium phosphate, pH 7.0, and 2 mM MgSO_4_) at 37°C until optical density at 600 nm (OD_600_) of ∼0.7 was reached. Expression of protein was induced for 16 h with 0.2 mM IPTG (isopropyl-β-d-thiogalactopyranoside) at 18°C. Cells were lysed in lysis buffer (25 mM HEPES, pH 7.6, 300 mM KCl, and 5% glycerol) containing lysozyme (1 mg/ml) and EDTA-free protease inhibitor tablets (cOmplete protease inhibitor; Roche). Cell suspension was sonicated 8 × 20 s at 27 to 30 W (power level, 3.5 to 4.0) with 40-s breaks between each pulse. Lysate was cleared by centrifugation in a 50-ml conical tube at 20,000 × *g* for 30 min (Fiberlite F13-14 × 50cy rotor in a Sorvall RC6 centrifuge). Supernatant was incubated with Ni-charged MagBeads (GenScript; catalog no. L00295) equilibrated with the binding buffer [25 mM HEPES, pH 7.5, 300 mM KCl, 5% glycerol, and 1 mM Tris(2-carboxyethyl)phosphine hydrochloride (TCEP)]. Bound proteins were washed with 50 ml binding buffer and eluted in elution buffer containing a gradient of imidazole ranging from 5 to 500 mM. Peak fractions were pooled and stored in 50% glycerol at −80°C.

### PAPD5 and PAPD7 ATP depletion assay.

Reactions were carried out in duplicate in 96-well low-profile, skirted, white plates (catalog no. AB0800WL; Thermo Fisher) where each well contained 10 μl of the reaction mixture consists of purified recombinant PAPD5 or PAPD7 proteins (12.5 nM) in 10 mM Tris-HCl (pH 8.0), 100 mM KCl, 5 mM MgCl_2_, 250 nM RNA substrate (CALM1) 5′-GCC UUU CAU CUC UAA CUG CGA AAA AAA AAA-3′, 750 nM ATP, 0.1 mM EDTA, 1 mM TCEP, and 0.002% NP-40. The reaction mixtures were incubated at room temperature for 3 h, and ATP depletion was monitored by using Kinase-Glo luminescent kinase kit following the manufacturer’s instructions (catalog no. V6712; Promega, WI, USA).

### Poly(A) tail length analysis of HBV transcripts.

The poly(A) tail of HBV transcripts was measured with the poly(A) tail-length assay kit (catalog no. 76455; Thermo Fisher) per the manufacturer’s instructions. Total RNA was added with poly(G/I) tail at the 3′ end and reverse transcribed by the primer specific to the poly(G/I) tail. HBV mRNA poly(A) tail was amplified by HBV-specific primer (5′-CAC CAG CAC CAT GCA ACT TT-3′, nucleotides [nt] 1806 to 1825 on HBV genome) and the universal primer that anneals to the G/I tail. HBV gene-specific PCR (GSP) was conducted using primers targeting nt 1633 to 1702 of HBV RNA with 5′-CCG AAT GTT GCC CAA GGT CT-3′ (sense) and 5′-CTC AAG GTC GGT CGT TGA CA-3′ (antisense). ACTB mRNA poly(A) tail was amplified by (sense) 5′-TTG CCA TCC TAA AAG CCA CC-3′ and the universal primer. ACTB GSP primers include 5′-CCC AGC ACA ATG AAG ATC AAG-3′ (sense) and 5′-GAC TCG TCA TAC TCC TGC TTG-3′(antisense). Amplified products were used as loading control. The obtained amplicon products were resolved on a 2% agarose gel. HBV poly(A) PCR products were sequenced on PacBio Sequel sequencing platform. Circular consensus sequencing (CCS) reads were generated from raw subreads using PacBio SMRTLINK software (v5.1). Accuracy cutoff default was 0.9. PCR primer sequences (allowing two mismatches) were used to identify segments potentially containing poly(A) tails in CCS reads. A CCS read could contain multiple such segments because of PCR conditions. These segments were processed separately in downstream analysis. Poly(A) tails were identified in each segment. Note: it was unsuccessful to sequence the sample “DMSO-treated WT (parent) cells” initially (Fig. 6J and K). In the second sequencing, two samples, including “DMSO-treated WT (parent) cells” and “DMSO-treated P7_KO (T2-22) cells,” were sequenced, and the sample “DMSO-treated P7_KO (T2-22) cells” served as a bridge to normalize two batches of sequencing data.

### HBV PRE *cis*-elements analysis.

Constructs containing either HBsAg or the *Gaussia* luciferase (Gluc) reporter genes were synthesized (GenScript). The H133 encodes the full HBsAg transcript sequence (spanning nt 2 to 1991; GenBank accession no. U95551) under the regulation of tetracycline-controlled cytomegalovirus (CMV) promoter. The H133_dSLα and H133_dLa are derived from H133 with either the SLα sequence (nt 1294 to 1322) or the La protein binding site (nt 1271 to 1294) deleted, respectively. In the luciferase-based constructs, the HBsAg encoding region was replaced with Gluc (the Gluc constructs). Variants were introduced into the Gluc constructs in which the HBx coding sequence was deleted with either wild-type SLα (Gluc_dHBx) or an inverted SLα sequence (Gluc_rcSLα). Huh-7 cells were transfected with the HBsAg or luciferase reporter-derived plasmids per the manufacturer’s instructions (Lipofectamine 3000; Invitrogen, MA, USA). Cells were treated with the indicated compounds for 5 days. Culture supernatants were used for HBsAg or luciferase measurement (Pierce Gaussia Luciferase Glow assay kit; Thermo Fisher Scientific, Waltham, MA, USA). Cells were collected for HBV RNA transcript and cellular rRNA analysis by Northern blotting.

### CRISPR-Cas9 knockout generation.

PAPD5 (GeneID 64282), PAPD7 (GeneID 11044), and ZCCHC14 (GeneID 23174) were knocked out with the CRISPR-Cas9 gene editing in the HepG2-hNTCP-C4 cells at GenScript Biotech Corporation (NJ, USA). Briefly, genomic RNAs (gRNAs) were designed and expressed in the plasmid pSpCas9 BB-2A-GFP PX458. The following three gRNAs were evaluated for each gene, and the gRNA with the highest cleavage efficiency was selected to knock out the target gene: PAPD5 gRNA (5′-GAC ATC GAC CTA GTG GTG TTT GG-3′), PAPD7 gRNA (5′-ATA TTT GGC AGC TTT AGT ACA GG-3′), and ZCCHC14 gRNA (5′-GCG TGA GAC CCG CAC CCC CG-3′). HepG2-hNTCP-C4 cells were transfected with validated gRNA-Cas9 plasmids. Transfected cells were sorted by fluorescence-activated cell sorter (FACS) through enhanced green fluorescent protein (EGFP). The obtained cell pool was expanded for single-cell cloning. Genomic DNA from the single-cell clones was extracted for PCR amplification with primers flanking the target site followed by Sanger sequencing.
